# Dihydromyricetin Reduced Bcl-2 Expression via p53 in Human Hepatoma HepG2 Cells

**DOI:** 10.1371/journal.pone.0076886

**Published:** 2013-11-04

**Authors:** Shixing Wu, Bin Liu, Qingyu Zhang, Jie Liu, Wei Zhou, Chang Wang, Mingyi Li, Shiting Bao, Runzhi Zhu

**Affiliations:** 1 Laboratory of Regenerative Medicine, Department of Hepatobiliary Surgery, Affiliated Hospital of Guangdong Medical College, Zhanjiang, China; 2 Emergency Department, Ningde Hospital, Affiliated Hospital of Fujian Medical University, Ningde, China; West German Cancer Center, Germany

## Abstract

Dihydromyricetin (DHM) is a major active ingredient of flavonoids compounds. It exhibited anticancer activity and induced apoptosis in human hepatocellular carcinoma HepG2 cells according to our previous data. In this study, we investigated whether p53 is involved in DHM-triggered viability inhibition and apoptosis induction in cancer cells. MTT [3-(4, 5-Dimethylthiazol-2-yl)-2, 5-diphenyltetrazolium bromide] assay was employed to evaluate the viability of HepG2 cells after DHM treatment. Meanwhile, p53 small interfering RNA (siRNA) was adopted to silence p53 expression. Protein level of p53 and Bax/Bcl-2 were evaluated by western blot analysis. Cell counting assay showed that DHM inhibited HepG2 cell growth effectively in a time- and dose-dependent manner. P53 expression was significantly increased after DHM treatment, whereas Bcl-2 was reduced potently. Furthermore, after co-treatment with Pifithrin-α (PFT-α, p53 inhibitor), Bcl-2 expression was reversed. The expression of Bax was no significant change, which was also observed after p53 silence. These findings defined and supported a novel function that DHM could induce human hepatocellular carcinoma HepG2 cells apoptosis by up-regulating Bax/Bcl-2 expression via p53 signal pathway.

## Introduction

**Figure 1:**
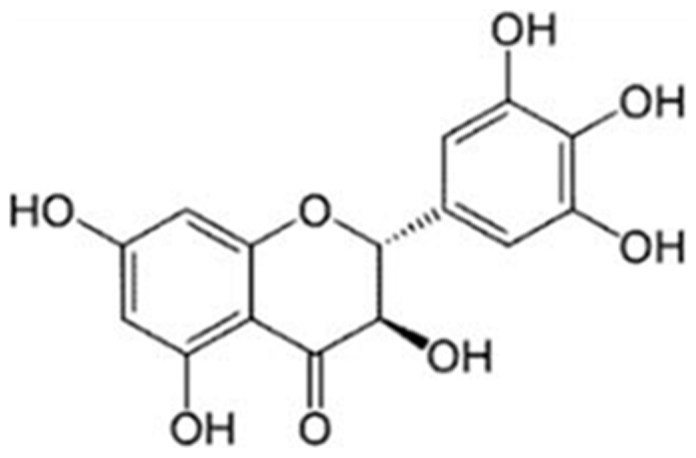
Chemical structure of DHM.

Dihydromyricetin (also named as Ampelopsin, Fig. 1) was isolated from the tender stem and leaves of the Ampelopsis grossedentata species, which widely distributed in South China. It was reported that the DHM can reach more than 30% in the tender stem and leaves of vine tea [1]. DHM has numerous pharmacological activities, such as anti-inflammatory, relieving cough, antimicrobial activity, anti-hypertension, anti-oxidation, hepatoprotective effect and anti-carcinogenic effect [2]–[5]. Recently, plenty of data supported that DHM could inhibit the growth and metastasis of prostate cancer *in vitro* and *in vivo* [6]. The existing data confirmed that DHM has a strong inhibitory activity against breast cancer MCF-7 cells and MDA-MB-231 cells, nasopharyngeal carcinoma HK-1 cells, liver cancer Bel-7402 cells, leukemia HL-60, K-562 cells and lung cancer H1299 cells [7]. Based on previous evidence, we focused on studying the correlation between p53 and Bcl-2 in human hepatocellular carcinoma HepG2 cells apoptosis induced by DHM.

P53 is a frequent target for mutation in human tumors, and mutant p53 proteins can actively contribute to tumorigenesis [8]. P53 functions as a cell nucleus phosphate protein for organizing whether the cell responds to various types and levels of stress with apoptosis, cell cycle arrest, senescence, DNA repair, cell metabolism, or autophagy [9]–[12]. As a transcriptional promoter, p53 protein could regulate a variety of target genes transcription and expression, thereby results in cell cycle arrest and apoptosis including Bcl-2, Bax, IGF1,p21WAF/CIP1, MDM2 and GADD45α. As is well known, Bcl-2, MDM2 and p21WAF1 have been considered as the direct or indirect targets for anticancer drug design [13]. The study of the expression pattern of p53 and Bcl-2 proteins in the hydatidiform moles showed a significant positive correlation between Bcl-2 and p53 [14]. Studies have shown that “Chk1-suppressed” pathway can be triggered in p53-deficient or Bcl-2-overexpressing human tumor cells [15]. In present study, it was found that gambogic acid which acts as an efficient apoptosis inducer could repress Bcl-2 expression via increasing p53 in MCF-7 cells [16]. Therefore, the aim of this study is to investigate the correlation of p53 and Bcl-2/Bax with DHM induced cell apoptosis in human hepatocellular carcinoma HepG2 cells.

**Table 1:** Primer sequences used for real-time quantitative PCR.

Gene sense	Anti-sense
*Bcl-2* ctttgagttcggtggggtca	gggccgtaca gttccacaaa
*TP53* ctcctcagcatcttatccgagt	gctgttccgtcccagtagatta
*Bax* aagctgagcgagtgtctcaag	caaagtagaaaagggcgacaac
*18s* cggcgacgacccattcgaac	gaatcgaaccctgattccccgtc

## Materials and Methods

### 2.1. Medicine and reagents

DHM was purchased from Sigma-Aldrich Inc, and it was solubilized in dimethylsulfoxide (DMSO) to a final stock concentration of 50 mM and stored at −20°C. Pifithrin-alpha (PFT-α), a p53 transcriptional inhibitor, was purchased from Sigma-Aldrich Inc. (St. Louis, MO). Antibodies anti-p53, Bcl-2, Bax and β-actin antibodies were obtained from Cell Signaling Technology (Beverly, MA). HRP-conjugated secondary antibody purchased from EarthOx (EarthOx, USA).LDH Cytotoxicity Assay Kit (Beyotime, China). P53 siRNA and its reference substance were purchased from Santa Cruz Biotechnology Inc. (Santa Cruz, CA). All these agents above-mentioned were diluted to the destined concentration. DMSO was used as a solvent control.

### 2.2. Cell lines and culture

Human hepatoma HepG2 cell line was offered by Affiliated Hospital of Guangdong Medical College (Zhanjiang, China). Cells were cultured in the RPMI 1640 medium (Gibco, Grand Island, NY), supplemented with 10% heat-inactivated fetal bovine serum (GIBICO, NY), penicillin 100 U/ml, and streptomycin 100 U/ml, and maintained in a humidified atmosphere of 95% air +5% CO_2_ at 37°C. HepG2 cells were grown in standard media, when the proliferation of cell is 70%–80% cells were treated with different concentrations of DHM.

### 2.3. Cell inhibition and cytotoxicity assay

MTT assay: Cell densities were adjusted to 2×10^4^ cells per 100 μL. Cells were seeded into a 96-well plate, which was placed in an incubator overnight to allow for attachment and recovery. Brieﬂy, cells were pretreated with 10, 25, 50, 75 and 100 μM DHM for 6, 12 and 24 h, MTT was dissolved at 5 mg/ml in warm assay medium. 20 μL of MTT solution was transferred to each well to yield a final volume 120 μL/well. Plates were incubated for 4 h at 37°C and 5% CO_2_. After incubation, supernatants were removed, and then 150 μL DMSO was added. The plate was placed on an orbital shaker for 5 minutes, and then the absorbance was recorded with reader (EnSpire™ 2300 Multilabel Reader, PE) at 595 nm. The principle is that the succinate dehydrogenase of live cells mitochondrial restores exogenous MTT to water insoluble violet crystal Formazan, which deposits in cells, but the dead cells have not this feature. Data were expressed as the percentage of viable cells as follows: inhibitory rate (%)  =  [A595 (control–A595 (treated))]/[A595 (control)–A595 (blank)] ×100%.

LDH assay: Cell densities were adjusted to ×10^4^ cells per 100 μL. Cells were seeded into a 96-well plate, which was placed in an incubator overnight to allow for attachment and recovery. Brieﬂy, cells were pretreated with 10, 50, 100 and 150 μM DHM for 24 h, and centrifuge the cells at 400 g for 5 min. Transfer 100 µl/well supernatant carefully into corresponding wells of an optically clear 96-well plate and add 100 µl Reaction Mixture to each well and incubate for up to 30 min at room temperature (Protect the plate from light). The absorbance was recorded with reader (EnSpire™ 2300 Multilabel Reader, PE) at 490 nm. Calculation of the Percentage Cytotoxicity: Cytotoxicity (%)  =  (Test Sample – Low Control)/(High Control – Low Control) X 100 [17].

### 2.4. Cell morphological assessment

Cells were dispersed to prepare the 1×10^6^ per mL cell suspension. After treatment with DHM and PFT-α (PFT-α treated HepG2 cells for 6 h, then treated with DHM for 12 h), cells were observed (100×) with inverted microscope (Leica, Wetzlar, Germany).

### 2.5. Apoptosis assay

Apoptotic cells were quantified using an Annexin V-FITC/PI kit (BioVision, CA, USA) and detected by ﬂow cytometry (FACSCalibur, Becton Dickinson), and analyzed by the software Modfit and CellQuest (BD Biosciences, Franklin Lakes, NJ, USA). The manual of the kit was strictly as follow [18]. Briefly, HepG2 cells were plated in the 6-well plates (1×10^5^ cells per well). 24 h later, the cells were treated with DHM (0, 10, 50, 100 μM) and incubated for 24 h. Then cells were collected, washed twice with cold D-hanks buffer solution, and resuspended in binding buffer (1×10^6^ cells/mL). After 100 μL of HepG2 cells was transferred to a tube, 5 μL of FITC-conjugated Annexin V (AnnexinV-FITC) and 5 μL of propidium iodide (PI) were added followed by incubation for 15 min at room temperature in the dark. The cells were set as positive depending on the fluorescence intensity of Annexin V-FITC or PI. In early stage of apoptosis cells were Annexin V positive; whereas Annexin V and PI positive cells were considered in the late stage of apoptosis.

### 2.6. P53 siRNA transfection

P53 siRNA and control siRNA were purchased from Santa Cruz Biotechnology Inc. Cells were seeded at a density of 1×10^6^ cells/mL on confocal dish. Cells were transfected at 80% confluence with the p53 siRNA plasmid and lipofectamine 2000^TM^ reagent (Invitrogen, San Diego, CA), according to the manufacturer's instructions. After 4 h, the medium was replaced by 1640 medium contained 10% FBS. The expression vector was transfected 24 h before treatment with different concentrations of DHM, cells with green spots were calculated under Leica fluorescence microscope (Leica, Wetzlar, Germany).

### 2.7. DHM regulated apoptotic proteins analysis

Western blotting analysis was performed using standard techniques as described previously [19]. Briefly, the cells were lysed in a lysis buffer (1 mL RIPA add 10 μL PMSF, Beyotime). Centrifuge the lysis cell sample for 10 min on high speed (13,000 rpm) at 4°C and the supernatant was collected. BCA Concentration measurement kit (P0012, Beyotime) was used for measuring the sample protein level, and then equal amounts of protein (50–200 µg/lane) were subjected to 12% sodium dodecyl sulfate-polyacrylamide gel electrophoresis (SDS-PAGE). Then proteins were transferred to polyvinylidene fluoride (PVDF, Millipore) and the membrane were blocked with 3% BSA for 1 h at 37°C and incubated with anti-Bcl-2, anti-p53 and β-actin overnight at 4°C. The membrance was incubated with appropriate HRP-conjugated secondary antibodies (1∶3000) for 1 h at 37°C, and washed with TBST 5×5 min. Chemiluminescent substrate (ECL, GE Healthcare) was added to the membrane and exposed strip from Kodak Image Station 4000 MM (USA).

### 2.8. Reverse transcriptase-polymerase chain reaction (RT-PCR)

According to the manufacturer's instruction, the total RNA was extracted with TRIzol® reagent (Invitrogen). RT-PCR was carried out by using an SYBR Premix Ex Taq (TaKaRa) with indicated primers (Table. 1), according to the instruction of LightCycler Real Time Cycler 480. Positive and negative controls were added. The PCR program: 95°C 4 min, 95°C 30 s, 60°C 40 s, 72°C 40 s, 12°C, forever, 40 cycles. After the reaction, the melt curves and amplification curves for all samples were recognized, then standard curves were made in Real-time quantitative PCR, meanwhile, as a housekeeping gene, 18S was utilized as an indicate.

### 2.9. Statistical analysis

The data was analysised by GraphPad Prism 5. All results shown represent means ± SD from triplicate experiments performed in a parallel manner unless otherwise indicated. Statistical differences were evaluated using the Student's t-test and considered significant at the * P<0.05, ** P<0.01 or *** P<0.001 level. All figures shown in this article were obtained from at least three independent experiments.

## Results

### 3.1. DHM inhibits cell proliferation and promotes cell apoptosis

**Figure 2:**
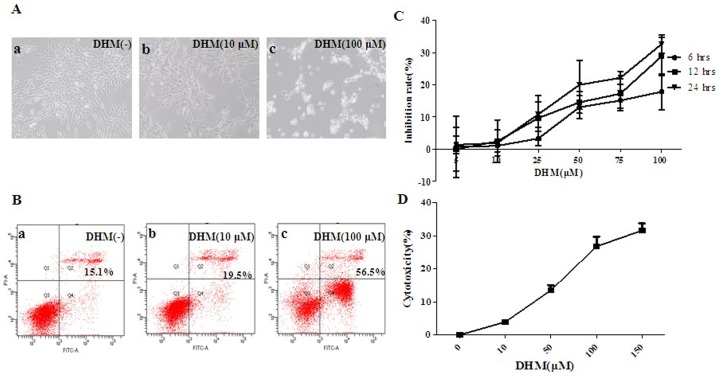
DHM inhibits HCC cells proliferation and promotes HCC cells apoptosis. (A) DHM-induced apoptosis in HepG2 cells with different concentrations (a: control, b: 10 μM, c: 100 μM) for 24 h, which was visualized by microscopy (×100). (B) DHM-induced apoptosis in HepG2 cells with different concentration ((0, 10, 100 μΜ) for 24 h, which was analyzed by ﬂow cytometry. (C) DHM-induced apoptosis in HepG2 cells with different concentrations (0, 5, 10, 25, 50 and 100 μΜ) for 6 h, 12 h and 24 h, which was analyzed by MTT method for cell growth inhibition rate. (D) Cytotoxicity of DHM was determined by LDH assay. Data demonstrated that DHM could affect HepG2 cells in a concentration dependent manner. Each sample was duplicated, and the figure is representative of three independent assays (n = 6). Values are means ± S.D. for at least three independent experiments performed in triplicate.

It was shown that untreated HepG2 cells grew well with clear skeletons, whereas cells treated with DHM were distorted, some of them became round and floating. The number of normal cells reduced, and sloughed cells increased in a concentration dependent manner. Annexin V/PI double staining assay method was performed to detect cell apoptosis (Fig. 2-A). Cell apoptosis was detected by Flow Cytometry, data shows DHM could induce cell apoptosis in a concentration-dependent manner (Fig. 2-B). MTT assay and LDH assay were used to evaluate the inhibitory effects and the cytotoxicity of DHM in HepG2 cells respectively. Data demonstrated that DHM could inhibit cell proliferation and promote apoptosis in human hepatocellular carcinoma HepG2 cells in time- and dose-dependent manner (Fig. 2-C, D). IC50 of DHM on HepG2 cells was 168 μΜ for 24 h treatment, which was calculated with GRAFIT-Erithacus IC50 software [20].

### 3.2. Cell growth recovered gradually after DHM withdrawal

**Figure 3:**
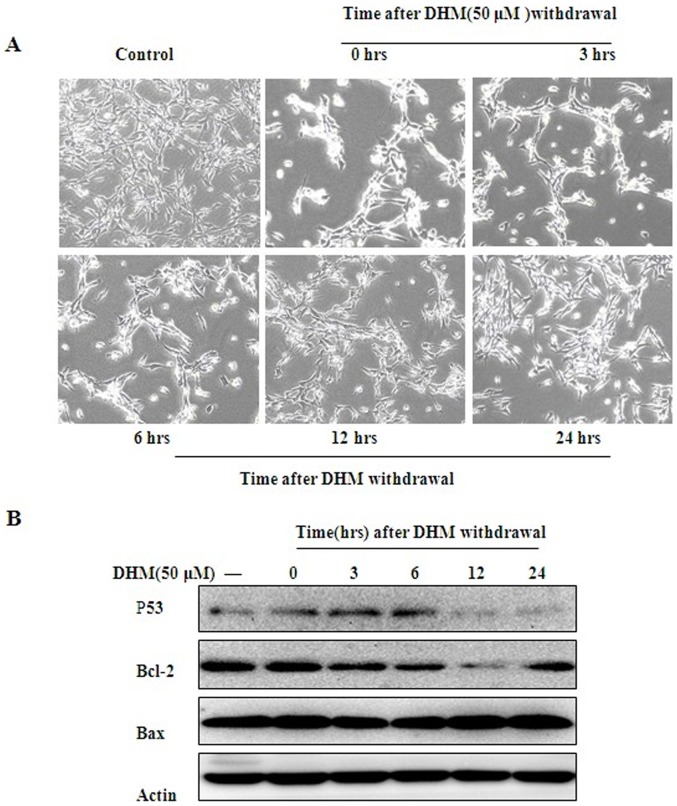
DHM function on the protein level of p53 and Bcl2. (**A)** Effect of DHM on HepG2 cell growth after DHM withdrawal. After administered to cells for 6 h, DHM was taken away and fresh culture medium was supplied. Cell growth was then detected at 3, 6, 12 and 24 h under the inverted microscope. (**B**) P53 and Bcl-2 protein levels after DHM withdrawal. After 6 h treatment, DHM was taken away and fresh culture medium was supplied. P53 and Bcl-2 proteins were detected at 0, 3, 6, 12, and 24 h by Western blot assay.

To confirm whether cell growth will be recovered after DHM withdrawal, HepG2 cells were treated with 50 μΜ DHM for 6 h thereafter replaced with fresh culture medium, and then cell growth was observed at 3, 6, 12 and 24 h after DHM withdrawal. Cells treated with 50 μΜ DHM for 6 h became round and floating, cell growth was inhibited and most HepG2 cells performed severe apoptosis (Fig. 3-A). 24 hours later, without DHM continuous treatment, cell growth recovered.

### 3.3. High levels of p53 were maintained up to 6 h after DHM withdrawal

In this study, we also evaluated p53 expressions after DHM withdrawal. Cells were treated with 50 μΜ DHM for 6 h and then supernatant was replaced with fresh culture medium. DHM increased p53 expression, which was maintained even after DHM withdrawal at 3 h and 6 h. However, with the extension of incubation time, p53 protein degradation was observed at 12 h and 24 h after DHM withdrawal. Meanwhile, Bcl-2 protein expression levels reduced after DHM withdrawal at 3 h, 6 h and 12 h. 24 hours later, with p53 decreased, Bcl-2 protein up-regulated. Bax protein expression levels changed insignificantly during the process. All the results strongly indicated that DHM could significantly regulate Bcl-2 protein via p53 (Fig. 3-B).

### 3.4. DHM inhibited Bcl-2 expression via p53 enhancement

**Figure 4:**
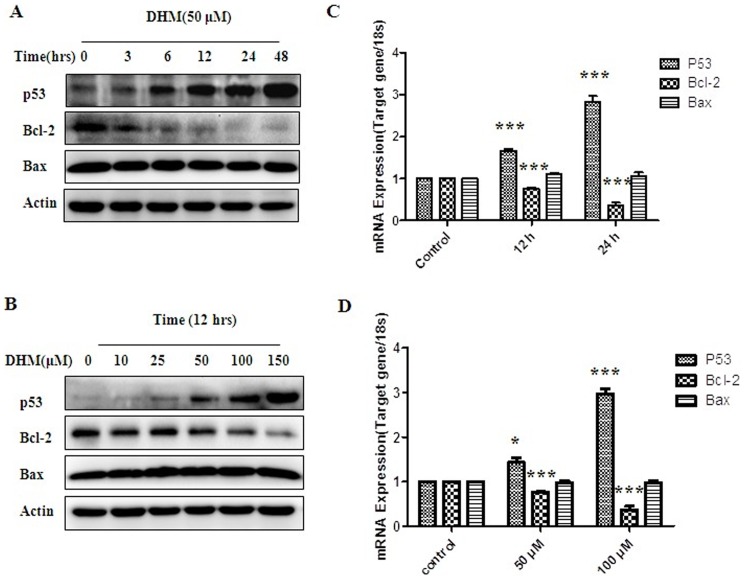
DHM affects the apoptotic gene expression. (A) Western blot analysis of proteins p53, Bcl-2 and Bax in HepG2 cells with DHM treatment for 3, 6, 12, 24 and 48 h. (B) HepG2 cells were treated with DHM (10, 25, 50, 100 and 150 μM) for 12 h. The correlation between p53 and Bcl-2 was shown by western blot assay. (C) Gene expressions of TP53, Bcl-2 and Bax in HepG2 cells treated with DHM for 12 h and 24 h, which were tested by RT-PCR assay. The expression of P53 are 1.8 and 2.8 folds higher than the control, meanwhile the expression of Bcl-2 are 0.8 and 0.4 folds lower than the control respectively. (D) Data shows the correlation between p53 and Bcl-2/Bax in HepG2 cells with DHM treatments of different concentrations for 24 h. The expression of P53 are 1.5 and 2.9 folds higher than the control, meanwhile, the expression of Bcl-2 are 0.8 and 0.5 folds lower than the control respectively. Each sample was duplicated, and the figure is representative of three independent assays(n = 6)(* P<0.05, ** P<0.01 or *** P<0.001).

Since DHM-trigged apoptosis is tightly associated with Bcl-2 related mitochondria-dependent apoptosis pathway, we further studied the correlation between p53 and Bcl-2 during DHM treatment. HepG2 cells were exposed to 50 μΜ of DHM for indicated period and the expression levels of p53 and Bcl-2 were evaluated. Fluorescence quantitative PCR results showed that the mRNA level expression of p53 increased and Bcl-2 reduced significantly with the increasing DHM concentration and treatment time, but the alteration of Bax was not obvious. (Fig. 4-C, D).

Meanwhile, p53 protein was up-regulated and Bcl-2 expression was reduced with the increasing DHM concentration and treatment time. After HepG2 cells were treated with 10, 25, 50, 100 and 150 μΜ of DHM for 12 h, p53 expression was up-regulated and Bcl-2 was reduced with the increase of DHM concentration. Cells were treated with 50 μΜ of DHM for 3, 6. 12, 24, and 48 h, p53 expression was up-regulated obviously and Bcl-2 reduced significantly with time-dependent manner, but the alteration of Bax expression was not obvious. However, levels of Bax/Bcl-2 proteins ratio in cells treated with DHM increased in a time- and dose-dependent manner. (Fig. 4-A, B)

### 3.5. P53 plays a key role in DHM-triggered apoptosis

**Figure 5:**
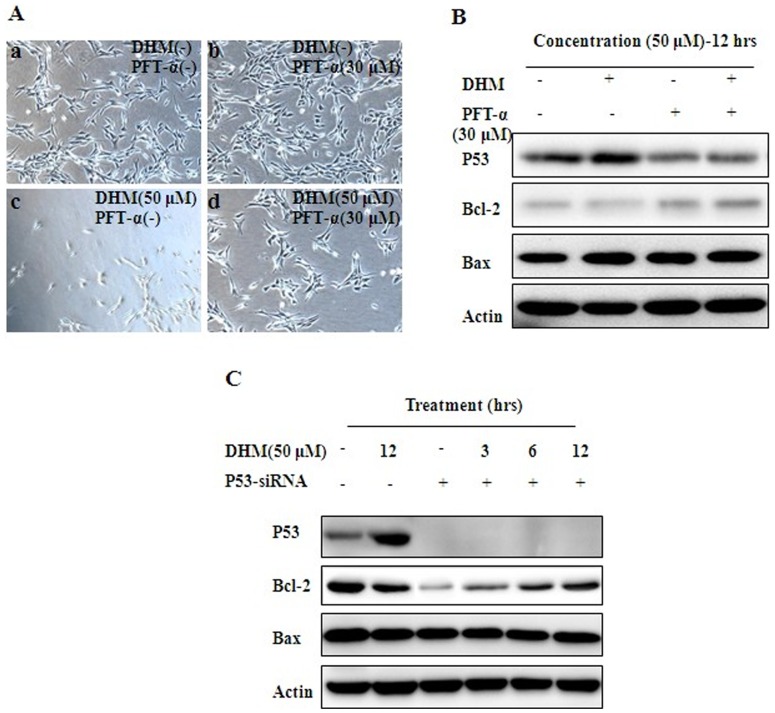
DHM induces HCC cells apoptosis by activation of p53 signaling. (A) Photographs show HepG2 cells were co-treated with 50 μM DHM or 30 μM PFT-α for 12 h. Cells exposed as following, a. DHM (−), PFT-α (−), b. DHM (−), PFT-α (30 μM), c. DHM (50 μM), PFT-α (−), d. DHM (50 μM), PFT-α (30 μM). (B) Western blot assay showes the correlation between p53 and Bcl-2 in HepG2 cells co-treated with 50 μM DHM and 30 μM PFT-α for 12 h. C P53 siRNA or control siRNA was transfected into HepG2 cells and then 50 μM DHM was administered for corresponding period.

DHM treatment lead to cell apoptosis in HepG2 cell line (Fig. 5-A). However, HepG2 cells were pre-treated with PFT-α for 6 h, and then DHM was added, cell apoptosis was alleviated. Moreover, p53 expression was suppressed by 30 μΜ of PFT-α (Fig. 5-B). Correspondingly, after p53 was knocked down by p53-siRNA, p53 protein decreased (Fig. 5-C), which implied the key role of p53 on DHM-induced apoptosis in HepG2 cells. With DHM-induced up-regulation of p53, Bcl-2 protein was decreased in a time-dependent manner.

## Discussion

DHM is known as an anticancer compound since it was characterized to be an apoptosis inducer [21], [22]. We found that DHM could inhibit HepG2 cell growth in a time- and concentration-dependent manner. In this study, we demonstrated that DHM induced human hepatoma HepG2 cells apoptosis by reducing Bcl-2 expression via p53 and highlighted the importance of p53 in DHM induced cell apoptosis.

High-throughput sequencing of cancer genomes, interestingly, uncovered the surprising fact that only a small number of mutated, deleted or amplified genes were discovered in sporadic cancers. The most frequently mutated genes independently from the origin of the tumor are the DNA damage checkpoint tumor suppressor genes *TP53* [23]. The apoptosis-inducing effect is more dependent on the balance of Bcl-2 and Bax than on Bcl-2 quantity alone [24], which plays a role in cell proliferation. Cell survival is steady if the expressions of Bcl-2 and Bax keep balance. The higher Bcl-2 expression level leads to inhibition of cell apoptosis [25], [26]. In our study, there are close connection between p53 and Bax/Bcl-2 in HepG2 cell apoptosis induced by DHM. Previous study demonstrated that ZnO nanorods induced apoptosis in human alveolar adenocarcinoma cells. The expressions of cell-cycle checkpoint protein p53 was up-regulated and the anti-apoptotic protein Bcl-2 was decreased [27]. Targeting the apoptosome in HepG2 cells by a cytotoxic component of Garcinia cowa, dulxanthone A has been reported. Dulxanthone A activated the intrinsic mitochondrial pathway via p53 upregulation, subsequent increase in Bax/Bcl-2 ratio, cytochrome c release, which induced apoptosome formation [28]. Notably, previous research confirmed that VX-680 increased Bax/Bcl-2 expression ratio, a favorable proapoptotic predictor for drug response and survival in AML [29].

When DHM was blocked by PFT-α or by p53 siRNA, here we found that DHM inhibited Bcl-2 expression via increasing p53 which was supported by the results that DHM modulated p53 before Bcl-2 and Bcl-2 repression was attenuated dramatically. Undoubtedly, p53 has been regarded as a comprehensive and valuable target in cancer research [30]. Recently it was reported that p53 was associated with cancer cell metastasis, metabolism, and small G protein signal transduction. As an apoptosis inducer, p53 could cause cell growth arrest, apoptosis, and senescence in response to various types of stimuli [31]–[34]. Consistent with our previous studies, we observed that DHM up-regulated p53 expression potently and this high expression level of p53 was maintained within 6 hours even after DHM withdrawal, indicating the long-lasting effect of DHM. Furthermore, DHM act as an efficient apoptosis inducer in HepG2 cells, could effectively increase Bax/Bcl-2 expression ratio via increasing p53. Previous studies demonstrated that the expression of Bcl-2 (anti-apoptotic protein) was significant to cell apoptosis. And the higher expression level of Bax/Bcl-2 proteins ratio in cells treated with DHM suggested that both p53 and Bax/Bcl-2 proteins also play a role in the pathway of DHM-induced apoptosis.
